# Barriers in access to healthcare services for chronic patients in times of austerity: an empirical approach in Greece

**DOI:** 10.1186/1475-9276-13-54

**Published:** 2014-07-25

**Authors:** Ilias-Ioannis Kyriopoulos, Dimitris Zavras, Anastasis Skroumpelos, Katerina Mylona, Kostas Athanasakis, John Kyriopoulos

**Affiliations:** 1 Department of Health Economics, National School of Public Health, 196 Alexandras Avenue, Athens 11521, Greece

**Keywords:** Barriers in access, Chronic patients, Unemployment, Income decrease, Economic crisis, Greece

## Abstract

**Objectives:**

To investigate the magnitude of barriers in access to health services for chronic patients and the socioeconomic and demographic characteristics that affect them.

**Methods:**

A cross-sectional study was conducted in 1,594 chronic patients suffering from diabetes, hypertension, COPD and Alzheimer. Logistic regression analyses were carried out in order to explore the factors related to economic and geographical barriers in access, as well as the determinants of barriers due to waiting lists.

**Results:**

A total of 25% of chronic patients face geographical barriers while 63.5% and 58.5% of them are in front of economic and waiting list barriers, respectively. Unemployed, low-income and low-educated are more likely to face economic barriers in access. Moreover, women, low-income patients, and patients with lower health status are more likely to be in front of geographical barriers. In addition, the probability of waiting lists occurrence is greater for unemployed, employees and low income patients.

**Conclusions:**

Barriers in access can be mainly attributed to income decrease and unemployment. In this context, health policy measures are essential for removing barriers in access. Otherwise, inequalities may increase and chronic patients’ health status will be deteriorated. These consequences imply adverse effects on health expenditure.

## Introduction

The concept of equal and universal access is acknowledged as a core priority for the egalitarian health systems [1]. It is noteworthy that access to healthcare is not merely defined in terms of service availability; Penchansky and Thomas defined the concept by analyzing the five dimensions of access, i.e. (a) availability, (b) accessibility, (c) affordability and (d) accommodation and (e) acceptability [2]. *Availability* is related to the adequacy of health resources, such as providers and facilities. *Accessibility* is associated with the location of the provision of healthcare and the location of patients, namely it includes concepts such as travel time and transportation cost. *Affordability* is related to the relationship between prices demanded by providers and the health insurance, ability to pay and income of patients. *Accommodation* is relevant to the organization in order the supply resources to be able to accept patients and serve their needs and expectations (e.g. hours of operation, telephone services). The last dimension, *acceptability*, is associated with some attributes of providers and patients (age, gender, ethnicity, religion etc.) and the impact of those on their attitude and behavior. Taking these aspects into consideration, the interaction between providers and patients may affect access to healthcare services.

In Greece, economic crisis and the implementation of austerity measures have worsened the self-rated health status as well as several health indicators [3,4]. The austerity measures have been associated with hospital mergers and reduction of hospital staff, having a negative impact on accessibility and availability. Moreover, reduction in disposable income decreased patients’ ability to pay, and therefore it negatively affected their affordability, with regards to healthcare services. In this context, economic crisis in Greece has negative impact on patients’ access to healthcare services [5,6]. By all means, this is not a Greek phenomenon, since historically economic crises have inflicted radical changes in the components of social protection, and, in specific, the access to healthcare services [7].

Access to adequate health care is an important aspect especially for patients suffering from chronic diseases, who constitute a group with many needs for healthcare and their number is high and increasing in plethora of developed countries [8]. Therefore, access and the difficulties occurring in times of economic crisis for chronic patients is a topic and a policy issue of great importance, given the impact of crisis on provision of healthcare and the increased vulnerability of chronic patients [9,10].

Based on these introductory comments, this paper aims to investigate the main characteristics related to economic and geographical barriers in access as well as barriers in access due to waiting lists for chronic patients, using data from the Greek healthcare setting, a system under severe constraints, austerity measures and ongoing consequences of a deep economic crisis.

## Methods

The analysis is based on primary data from a sample consisting of 1,594 chronic patients suffering from Alzheimer, hypertension, diabetes and chronic obstructive pulmonary disease (COPD). These patients were participants in a cross-sectional study that was conducted in March 2013. Patients were approached through their treating physician. In more detail, 160 physicians were randomly selected through the official list of the National Medical Association of Greece, based on specialty and geographical distribution. Primary care physicians comprised 27.5% of the sample of the physicians. The selected physicians were requested to provide the contact details of 10 patients suffering from the aforementioned chronic diseases. Specifically, each physician was provided with a set of 10 random numbers between 1 and 20. According to those numbers, physicians were asked to inform 10 of their patients that visit their practice about the objectives of the study and to provide them with the patient consent form. Ethical approval was obtained from the Hellenic Data Protection Authority (reference number 1139) and from the Bioethics Committee of the National School of Public Health.

As a second step, the research team approached the patients and derived the study data via telephone interviews, based on a structured questionnaire. Participants answered a series of closed type questions, including self-rated health status (currently and one year before the interview), income (current and change during the last year), educational level and occupation. The respondents were 394 patients suffering from Alzheimer, 400 patients suffering from hypertension, 400 patients suffering from diabetes type II and 400 patients suffering from chronic obstructive pulmonary disease (COPD). The specific diseases were chosen on grounds of epidemiology/disease burden [11] as well as their socioeconomic impact on the Greek healthcare system [12-15]. The sample size was determined by the prevalence of each condition with an expected response distribution of 50% and predefined acceptable margin error of 5% and 95% confidence interval. According to the above each disease group sample was constituted by 400 patients. The percentage of patients that refused to participate in the study was 33.6%. Table 1 presents a more detailed description of the sample.

**Table 1:** Sample characteristics

**Gender**	
Male	632
	(39.6%)
Female	962
	(60.4%)
**Age**	
<50	461
	(28.9%)
51-60	445
	(28.0%)
61-70	355
	(22.2%)
>71	333
	(20.9%)
**Area**	
Urban	1,042
	(65.4%)
Rural	552
	(34.6%)
**Total**	1,594

A series of ordinal logistic regression models were constructed, in order to explore the impact of a set of baseline parameters of the population to the dependent variables of interest. In this paper, three different models are presented, each of them focusing on the impact of socioeconomic variables on the economic barriers, the geographical barriers and the barriers due to waiting lists as dependent variables.

Income, educational level, occupation, age, health status and gender were used as independent variables of the aforementioned regression models. The choice of these regressors can provide a wide framework of the socioeconomic status and the demographic and health characteristics. The measurement of these variables is analytically presented in Table 2. The analyses were conducted in the STATA 09 statistical software.

**Table 2:** Measurement of variables

**Value**	**Barriers (economic, geographic, waiting lists)**	**Income (€)**	**Educational level (number of observations)**	**Occupation (number of observations)**
1	Very easy	No income	No education/illiterate (49)	Employer (132)
2	Easy	1-300	Attended primary/elementary school (362)	Employee (387)
3	Neutral	301-500	Attended junior high school (150)	Employee without wage (e.g. in family firm) (10)
4	Difficult	501-750	Attended a technical school (35)	Unemployed (134)
5	Very difficult	751-1,000	Attended a senior technical school (31)	Retired (699)
6	Don’t know/don’t answer	1,001-1,500	Attended a senior high school (431)	Housewife (207)
7		1,501-2,000	Attended a private college after senior high school (98)	Student (5)
8		2,001-3,000	Attended a technical institution after high school (134)	Other (17)
9			Attended a university (282)	Don’t know/don’t answer
10			Studied at postgraduate level (27)	
11			Don’t know/don’t answer	

Note: Age was directly derived from the date of birth. Self-rated health status (from 0: close to death to 100: excellent health) was used to measure health status. Gender was measured as 1: male and 2: female.

## Results

Self-rated health status of chronic patients was deteriorated between 2012 and 2013 (62.2% in 2012 vs. 58.5% in 2013). This fact is more intense for the patients suffering from Alzheimer (53.9% in 2012 vs. 47.0% in 2013), while the deterioration of self-rated health is the lowest for patients suffering from diabetes (63.6% in 2012 vs. 61.7% in 2013). Moreover, chronic patients mentioned a significant income decrease and an expenditure reduction in many aspects related to their lifestyle. However, the reported drop of the expenditure for health and education is much lower than the corresponding for other needs. It is noteworthy that the reported income decrease is approximately 34.1% (1668.5 in 2012 vs. 1099.2 in 2013), while the private health expenditure is estimated at 10.2% of the total family income. Furthermore, 24.8% of the interviewees face increased difficulties in access to healthcare services due to geographical barriers, while the corresponding percentage for the economic barriers is approximately 62.8%. In addition, 55.1% of the respondents are in front of increased difficulties in access because of the presence of waiting lists.

The aforementioned descriptive aspects provide a general framework, or a "big picture" of the access to healthcare services for chronic patients. However, apart from mentioning the problem, it is crucial to search for more specific characteristics. In this context, some ordinal logistic regressions were carried out in order to illustrate the characteristics which lead to increased probability of barriers occurrence. The results are represented in Tables 3, 4 and 5.

**Table 3:** Ordinal Logistic Regression of economic barriers in access to healthcare services

**Access economic**	**Coef.**	**Std. Err.**	**Z**	**p > |z|**	**95% Conf. interval**
Income	-0.198	0.036	-5.53	0.000	-0.269, -0.128
Education	-0.040	0.020	-1.97	0.049	-0.079, -0.000
Occupation 2	0.242	0.180	1.35	0.178	-0.111, 0.596
Occupation 3	0.674	0.577	1.17	0.243	-0.457, 1.806
Occupation 4	0.550	0.229	2.40	0.016	0.100, 0.999
Occupation 5	0.174	0.176	0.99	0.322	-0.170, 0.519
Occupation 6	0.130	0.206	0.63	0.528	-0.273, 0.534
Occupation 7	-0.866	0.813	-1.06	0.287	-2.459, -0.728
Occupation 8	0.734	0.530	1.38	0.166	-0.305, 1.773

2013. Greece.

Log Likelihood = -2338.664.

Number of Obs = 1586.

LR chi2(9) = 69.98.

Prob > chi2 = 0.0000.

Pseudo R2 = 0.0147.

Note: Income, educational level, occupation, age, health status and gender were all used as independent variables of the models. The analysis considered the effect of the aforementioned variables, in order to avoid the possibility of confounding. We have included these variables in the models and evaluated whether they were potential confounders. We did not present the betas for all these variables because they were not statistically significant.

**Table 4:** Ordinal Logistic Regression of geographical barriers in access to healthcare services

**Access geographic**	**Coef.**	**Std. Err.**	**z**	**p > |z|**	**95% Conf. interval**
Income	-0.127	0.035	-3.60	0.000	-0.196, -0.058
Occupation 2	0.674	0.209	3.23	0.001	0.265, 1.083
Occupation 3	1.457	0.553	2.63	0.008	0.373, 2.541
Occupation 4	0.626	0.248	2.53	0.012	0.140, 1.112
Occupation 5	0.626	0.199	3.15	0.002	0.237, 1.015
Occupation 6	0.625	0.231	2.71	0.007	0.172, 1.077
Occupation 7	2.041	0.861	2.37	0.018	0.354, 3.729
Occupation 8	1.005	0.551	1.82	0.068	-0.075, 2.084
Gender	0.223	0.103	2.17	0.030	0.021, 0.424
Health	-0.016	0.002	-7.03	0.000	-0.021, -0.012

2013. Greece.

Log Likelihood = -2166.146.

Number of Obs = 1571.

LR chi2(10) = 101.87.

Prob > chi2 = 0.0000.

Pseudo R2 = 0.0230.

Note: Income, educational level, occupation, age, health status and gender were all used as independent variables of the models. The analysis considered the effect of the aforementioned variables, in order to avoid the possibility of confounding. We have included these variables in the models and evaluated whether they were potential confounders. We did not present the betas for all these variables because they were not statistically significant.

**Table 5:** Ordinal Logistic Regression of barriers in access to healthcare services due to waiting lists

**Access waiting lists**	**Coeff.**	**Std. Err.**	**Z**	**p > |z|**	**95% Conf. interval**
Income	-0.093	0.034	-2.77	0.006	-0.159, -0.027
Occupation 2	0.469	0.182	2.58	0.010	0.113, 0.826
Occupation 3	0.698	0.618	1.13	0.259	-0.513, 1.909
Occupation 4	0.531	0.231	2.29	0.022	0.077, 0.984
Occupation 5	0.283	0.173	1.64	0.102	-0.056, 0.621
Occupation 6	0.321	0.205	1.57	0.117	-0.080, 0.722
Occupation 7	-0.060	0.843	-0.07	0.943	-1.712, 1.592
Occupation 8	-0.236	0.468	-0.50	0.615	-1.153, 0.682

2013. Greece.

Log Likelihood = -2360.4172.

Number of Obs = 1573.

LR chi2(9) = 19.05.

Prob > chi2 = 0.0146.

Pseudo R2 = 0.0040.

Note: Income, educational level, occupation, age, health status and gender were all used as independent variables of the models. The analysis considered the effect of the aforementioned variables, in order to avoid the possibility of confounding. We have included these variables in the models and evaluated whether they were potential confounders. We did not present the betas for all these variables because they were not statistically significant.

The economic barriers in access depend on income [Coeff. -0.20, C.I. (-0.27,-0.13)] and educational level [Coeff. -0.04, C.I. (-0.08, 0.00)]. Moreover, unemployment is also an important aspect which causes economic barriers in access to healthcare services [Coeff. 0.55, C.I. (0.1, 1.00)]. It is noteworthy that both the coefficients of income and educational level have a negative sign. This means that a higher income or educational level leads to lower probability of occurrence of economic barriers. It is noteworthy that gender and health status do not have impact on the difficulties in access due to economic aspects.

Another type of barriers in access is related to difficulties due to geographical reasons. Specifically, low-income patients are more likely to face increased difficulties in access to healthcare services for chronic patients [Coeff. -0.13, C.I. (-0.20, -0.06)]. Moreover, women [Coeff. 0.22, C.I. (0.02, 0.42)] and patients with lower health status [Coeff. -0.02, C.I. (-0.02, -0.01)] are more likely to face geographical barriers in access. According to the analysis of this paper, the type of occupation has also impact on the access of chronic patients, through the geographical difficulties that may occur. It is noteworthy that almost all occupational categories demonstrate a statistically significant relationship with geographical barriers occurrence. However, students are more likely to be in front of geographical barriers, compared with other occupational categories. Employees without wage will probably face less geographical barriers than the students, but more than the typical employees, the unemployed, the housewives and the retired chronic patients. Furthermore, the ordinal logistic regression for this case does not conclude that education is a statistically significant regressor.

Barriers in access due to waiting lists represent the difficulties that occur because of the cost of time. High-income chronic patients will probably face fewer barriers due to waiting lists [Coeff. -0.09, C.I. (-0.16, -0.02)]. In addition, some specific occupational categories have a statistically significant relationship with the probability of waiting lists barriers occurrence. These are the employees [Coeff. 0.45, C.I. (0.09, 0.80)] and the unemployed patients [Coeff. 0.51, C.I. (0.06, 0.96)].According to this regression the unemployed patients are more likely to face barriers in access due to waiting lists than the employed ones. Moreover, education, gender and health status do not consist statistically significant determinants of barriers in access due to waiting lists.

## Discussion

According to the results of this analysis, income level is a major factor that affects access to healthcare services. High-income patients are able to overcome the barriers in access, as they can afford to pay for the quest of healthcare outside the public healthcare services frame, implying that high income can increase the alternatives, such as private services or, even, informal payments. It is noteworthy that high private spending and high informal payments are both typical characteristics of the Greek health system [16,17]. Thus, these alternatives are consistent with the general framework of healthcare services in Greece. Geographical barriers and barriers due to waiting lists are also inevitable for low-income chronic patients, because the alternatives are not affordable for them.

The educational level was also an independent variable of the regressions of this study, as it is regarded as a crucial determinant of socioeconomic status. Generally, higher prevalence in chronic diseases is observed in the population group characterized by low education [18]. Moreover, several findings suggest that people with higher education are less likely to suffer from chronic diseases [19]. As shown previously, a high educational level has impact on economic barriers in access, but not in the other types of barriers. A first explanation for this claim is related to an indirect link, namely that education increases the probability of finding a good and well-paid job. Therefore, education leads to higher income and consequently to fewer barriers in access. However this claim is inadequate to explain the findings to their full extend, as a higher income also implies fewer geographical barriers and barriers due to waiting lists. Our empirical results do not verify this claim, because education does not have impact on geographical and waiting lists barriers. Another approach is associated with the claim that well-educated patients have better information about the alternatives of their treatment and they are more aware about health promotion and prevention [19]. Therefore, they can overcome the potential economic barriers in access through self-monitoring and better information about the alternatives. Moreover, another aspect that affects access to healthcare services is related to social networks. Specifically, patients with high educational level have a stronger and more influential social network [20], which may reduce the economic barriers and facilitate access to healthcare.

The type of occupation has also impact on the barriers in access to healthcare services. Specifically, unemployed chronic patients are more likely to face economic barriers in access, mainly because unemployed have very low income. This finding is quite reasonable, given the high unemployment rate in Greece during the recession of the last years. It is noteworthy that unemployment also causes barriers in access due to waiting lists, partly because the low income reduces the alternatives, such as private healthcare. Moreover, geographical barriers are more likely to occur in students, and in self-employed in the family firm.

Although barriers in access to healthcare services are identified by many aspects, the main drivers which cause these difficulties are directly related to economic recession and the current restrictive fiscal policy. Specifically, low income and unemployment are the key characteristics affecting difficulties in access.

Other studies conclude to similar results, as socioeconomic status (namely income, education and occupation) affects chronic diseases prevalence. Specifically, chronic diseases are more prevalent in the lower socioeconomic groups [21]. Moreover, the features of socioeconomic status are associated with disparities in health status and differences in access to healthcare services [22,23].

Despite the ongoing burden of chronic diseases and the implications in access of chronic patients, health policies are not oriented toward facing and managing them [24,25]. Nowadays, the reconsideration of health agenda through the prioritization of chronic diseases management is a crucial challenge for every health system [8,25,26]. Generally policy interventions are necessary not only from a public health perspective but also from an economic point of view, and they can be beneficial in terms of efficiency and equity [27]. In his influential paper, Kenneth Arrow provided economic rationale for government intervention in healthcare sector [28], which is relevant to chronic diseases as well. In this framework, economic theory implies that it is reasonable for a government to intervene, as chronic diseases and their risk factors are associated with market failures, such inadequate and asymmetric information, externalities and time-inconsistent preferences [29].

A first dimension of a health policy plan for chronic diseases is associated to public health and prevention actions, namely that government, health organizations and NGOs should inform the population about the risk factors, (e.g. diet, physical activity and lifestyle). However, although prevention of chronic diseases is a low-cost and effective solution, political will and support towards this direction is limited [30]. The other dimension of a strategy for chronic diseases is related to their management and the search for optimal care model. Many chronic diseases can be managed through patients’ regulation, self-management and systematic monitoring by GPs and primary care [31-34]. The development of a care model based on these aspects can reduce the needs for systematic specialty care, which is more expensive and less accessible.

Despite the importance of lifestyle and risk factors, our analysis indicates that economic and social causes are the main factors affecting barriers in access. Therefore, the aforementioned actions (namely prevention actions and chronic diseases management) should be accompanied with interventions that face the main drivers of barriers in access. In this context, social and health policies are considered as essential actions for the decrease of the adverse impact of unemployment and low income on patients’ access. Such actions are becoming more urgent, in a period of economic crisis. Therefore, a social safety net against the increasing unemployment and the income decrease could improve patients’ access, by reducing the reported barriers. These actions constitute an integral part of the agenda, and thus they should be transformed into specific policy actions for the vulnerable groups of the population.

As with any study of this kind, the present one also has some limitations that should be acknowledged. Ideally, the sample would consist of chronic patients throughout the whole chronic diseases spectrum. Unfortunately, this study selected to examine four chronic diseases, due to resource constraints. Moreover, we could not select the interviewees from a survey that is based on the total population, as it was costly and time-consuming. Thus, we chose the aforementioned methodology. In addition, this study focuses on the barriers in access in a period of economic recession and crisis. Although it would be interesting to present the determinants of barriers in access, based on the whole period of an economic cycle, we think that this approach is useful in terms of health policy. This claim is based on the fact that the abovementioned finding could be taken into consideration when governments decide to implement restrictive fiscal policy and austerity measures either by "chance" (that is, due to an economic crisis) or by "choice".

## Conclusions

Income has significantly fallen since 2010 and supply has been reduced in healthcare sector. In this context, according to this analysis, economic crisis has led to barriers in access to healthcare services for chronic patients, who constitute a vulnerable group of the population with increased needs for healthcare. Income drop and unemployment –both consequences of the economic crisis- constitute the main reasons of the difficulties in access.

In this framework, health policy should take into consideration chronic diseases management, in order to reduce the consequences not only in terms of costs (which are high and increasing) but also for the accessible provision of healthcare to chronic patients. Therefore, although health policy is not oriented towards chronic diseases management and their risk factors, a reconsideration of the agenda in Greece would be beneficial in terms of access, public health and potential savings. Moreover, social policy measures -for supporting chronic patients who face difficulties- are essential, during a period in which the adverse effects of the main drivers of barriers in access are increasing.

### Consent

Written informed consent was obtained from the patients for the publication of this report.

## Competing interest

The authors declare that they have no competing interests.

## Authors’ contributions

DZ, AS and KM contributed to the conception and the design of the study and the acquisition of the data. IIK, DZ, AS, KM and KA contributed to the analysis and the interpretation of the data. IIK, DZ and KA drafted the manuscript. JK contributed to the critical revision of the manuscript for important intellectual content. All authors read and approved the final manuscript.
